# Is the dolphin a fish? ERP evidence for the impact of typicality during early visual processing in ultra-rapid semantic categorization in autism spectrum disorder

**DOI:** 10.1186/s11689-022-09457-7

**Published:** 2022-08-23

**Authors:** Ann-Kathrin Beck, Daniela Czernochowski, Thomas Lachmann, Bernardo Barahona-Correa, Joana C. Carmo

**Affiliations:** 1 Center for Cognitive Science, University of Kaiserslautern, Kaiserslautern, Germany; 2 Centro de Investigación Nebrija en Cognición, Universidad Nebrija, Madrid, Spain; 3 University of Leuven, Leuven, Belgium; 4 Champalimaud Research & Clinical Centre, Champalimaud Foundation, Lisboa, Portugal; 5 Faculdade de Ciências Médicas, NOVA Medical School, Universidade Nova de Lisboa, Lisboa, Portugal; 6 CADIn – Neurodesenvolvimento e Inclusão, Cascais, Portugal; 7 Faculdade de Psicologia, Centro de Investigação Em Ciências Psicológicas, Universidade de Lisboa, Lisboa, Portugal

**Keywords:** Autistic spectrum, High functioning, EEG, ERP, D prime, Presentation time

## Abstract

**Background:**

Neurotypical individuals categorize items even during ultra-rapid presentations (20 ms; see Thorpe et al. Nature 381: 520, 1996). In cognitively able autistic adults, these semantic categorization processes may be impaired and/or may require additional time, specifically for the categorization of atypical compared to typical items. Here, we investigated how typicality structures influence ultra-rapid categorization in cognitively able autistic and neurotypical male adults.

**Methods:**

Images representing typical or atypical exemplars of two different categories (food/animals) were presented for 23.5 vs. 82.3 ms (short/long). We analyzed detection rates, reaction times, and the event-related potential components dN150, N1, P2, N2, and P3 for each group.

**Results:**

Behavioral results suggest slower and less correct responses to atypical compared to typical images. This typicality effect was larger for the category with less distinct boundaries (food) and observed in both groups. However, electrophysiological data indicate a different time course of typicality effects, suggesting that neurotypical adults categorize atypical images based on simple features (P2), whereas cognitively able autistic adults categorize later, based on arbitrary features of atypical images (P3).

**Conclusions:**

We found evidence that all three factors under investigation — category, typicality, and presentation time — modulated specific aspects of semantic categorization. Additionally, we observed a qualitatively different pattern in the autistic adults, which suggests that they relied on different cognitive processes to complete the task.

## Introduction

Imagine a dolphin — based on its fins or its habitat in the water, it could be classified as a fish; however, unlike other marine animals, it belongs to the category of mammals. While this particular common misconception contradicts a rule-based biological taxonomy, some semantic categories remain subjective, as multiple (hierarchical) levels of categorical membership are possible. Notably, individuals on the autistic spectrum have been shown to prefer more specific, local features for categorization [51]. In this study, we investigate how neurotypical adults and those on the autism spectrum categorize typical vs. atypical exemplars of two semantic categories (animals and food). We focus on the temporal order of categorization processes by using event-related potentials (ERPs) to investigate early processing stages of semantic categorization of visually presented stimuli.

### Neural activity during semantic categorization within the first 300 ms

Neural activity as measured by ERPs during categorization distinguishes between several categorization processes very early on. The first modulation is observed in the N1 component (about 140 ms after target presentation at fronto-central electrodes) mirroring different levels of categorization. Visual input can be semantically categorized either on a general, superordinate level (e.g., “animal”), on a less general, basic level (e.g., “dog”), or on a more detailed, subordinate level (e.g., “poodle”; [29]. Tanaka, Luu, Weisbrod, and Kiefer [63] found that N1 was larger when images were categorized based on the subordinate level compared to categorizations on the basic or superordinate level. The authors concluded that subordinate categorization requires more perceptual processing. A second ERP modulation, the anterior P2, is observed when targets are discriminated based on simple features [35]. When categorization is more difficult, P2 amplitude (between 190 and 240 ms) has been found to be smaller [8]. A third ERP component is the anterior N2 (between 240 and 300 ms) indexes, among other cognitive processes, categorization and object recognition [69]. As part of the brain’s action monitoring system, N2 is enhanced by conflict eliciting stimuli, for instance those difficult to classify [71], but is not affected by categorization levels (superordinate, basic, or subordinate [40] for review, see [18]. Finally, the P3 occurs when distinguishing complex, sometimes arbitrary target features [35] and has been associated with memory and attentional processing leading to event classification [31, 55]. Proverbio, Del Zotto, and Zani [52] asked participants to decide whether pairs of stimuli representing animals and/or man-made objects belonged to the same category. A larger centro-parietal P3 component was observed for animals compared to objects, even when salience was controlled for, suggesting smaller processing demands for animal recognition or greater involvement of visual sensory areas responsible for distinguishing complex features [52].

### Ultra-rapid semantic categorization

Under time pressure as implemented by ultra-rapid stimulus presentation (~ 20 ms), the earliest difference in ERP components related to semantic categorization was found 150 ms after stimulus onset over the frontal and occipital cortex (ultra-rapid categorization; first investigated by Thorpe, Fize, & Marlot, [64]). In this paradigm, participants are asked to release a button if they see an animal image (“go” trials) and to keep their finger on the button if the image does not represent an animal (“no-go” trials). The difference between animal and non-animal images (dN150) was characterized by a positive peak at 186 ms over frontal electrode sites. Using this paradigm, similar results were reported for animals and vehicles [67], animal and non-animal pictures (i.e., mountains, rivers, buildings, fruits, and vehicles; [3], man-made and natural objects [27], and with extensively trained but newly established categories [13]. Together, these results suggest that ultra-rapid semantic categorization is based on a coarse visual representation [14].

### Categorization in autism spectrum disorder

In autism spectrum disorder (ASD), the semantic system, including semantic categorization, has not received much attention. However, differences in the mechanisms underlying categorization may contribute to the pattern of social, communication, and behavioral characteristics of ASD [21]. For instance, during speech acquisition, infants form categories of sounds. If this ability is impaired in infants, secondary difficulties in acquiring speech may arise, and indeed impaired or delayed development of communicative speech is one of the main characteristics of many on the autistic spectrum (e.g., [62]).

Prior research on the semantic categorization of pictorial information in ASD has predominantly relied on indices of behavioral performance (for neuroimaging findings during word categorization, see, e.g., [19]. Based on the paradigm by Thorpe and colleagues [64] detailed above, Vanmarcke et al. [66] explored differences between autistic and not-autistic adults, depending on categorization levels (superordinate, basic, and subordinate). In line with the behavioral findings of Tanaka and colleagues [63], Vanmarcke et al. [66] found differences between levels of categorization in both groups. Of particular relevance for the present study, they did not observe any behavioral differences between autistic and neurotypical (NT) adults. However, this study does not provide information about the mechanisms underlying semantic categorization. One hint regarding the mechanisms underlying semantic categorization may be found in a study by Carmo et al. [7]. In that study, individuals with and without a diagnosis of ASD participated in a dot-pattern matching task. They performed either an identity matching task (the patterns are the same in shape and orientation) or a category matching task (the patterns are the same in shape but of different orientation). They observed, in both tasks, a group effect, with slower reaction times (RTs) for autistic compared to NT adults. This result suggests overall slower category learning in autistic individuals [7]. Minshew, Meyer, and Goldstein [44] suggest that cognitively able autistic adults categorize items based on simple, rule-based features but seem to have difficulties when distinguishing input based on more complex, less perceptually apparent features. In line with this, Gastgeb and Strauss [21] found that cognitively able autistic adults have difficulties in forming abstract categorical prototypes, due to enhanced discrimination and reduced generalization. Together, this pattern of results suggests that the mechanisms underlying semantic categorization are qualitatively different in autistic individuals.

### Typicality in ASD

Since most natural categories have no distinct boundaries, they are not distinguished based on simple features but rather based on “typicality structures” [21]. Recent behavioral studies suggest that cognitively able autistic individuals have difficulties with the outer edges of a category [5, 20]. In more detail, some members of a category are more representative and therefore more *typical* for a specific category (e.g., sparrow as a bird) than other, less representative and therefore *atypical* members (e.g., ostrich as a bird). Gastgeb, Strauss, and Minshew [20] investigated how typicality structures influence categorization of an artificial (e.g., furniture) and a natural (e.g., animal) category in cognitively able autistic adolescents. Responses for both categories were slower and more error-prone for atypical than for typical items in cognitively able autistic and NT adolescents, showing that atypical stimuli require additional processing — attentional, perceptual, memory related, or decision related (or multiple) — in order to be categorized. However, whereas cognitively able autistic individuals processed typical exemplars as efficiently as NT adolescents, autistic individuals required additional processing for atypical exemplars, in line with the notion that categorization on a superordinate level can occur without detailed visual processing, whereas both basic and subordinate categorization rely on further perceptual information. Thus, atypical items need to be categorized on a more detailed level than typical items [26]. To examine this in more detail, Carmo et al. [6] presented six images in a rapid-serial visual presentation paradigm with different presentation times (13, 27, 53, and 80 ms). Participants were asked to identify a target item belonging to a basic-level category (typical and atypical mammals, birds, vehicles, and fruits). Performance was strongly affected by typicality, with a higher detection rate for typical items. Atypical items were only detected by NT adults in the condition with the longest presentation time, since the extraction of more perceptual information requires additional processing. Contrary to NT participants, cognitively able autistic participants were not able to detect atypical items even in the condition with the longest presentation time, suggesting qualitative differences in categorization. Together, these results suggest that there is an interaction effect between typicality and presentation time which affects semantic categorization in autistic individuals.

### The present study

To the best of our knowledge, to date, no study combined all potentially relevant and interplaying aspects introduced above. Hence, in the present study, we assessed typicality effects in categories with different distinctiveness of their category boundaries in ultra-rapid categorization with different presentation times in cognitively able autistic and NT adults using EEG. We adapted the paradigm introduced by Thorpe et al. [64] and tested a sample of cognitively able autistic participants and a NT control group matched for age, schooling, and general cognitive abilities. To investigate the influence of category boundaries in cognitively able autistic individuals (based on [44]), we added a second semantic category (food) with even less distinct boundaries than animals and presented the stimuli with two different presentation times. While the presentation time less influences the processing of typicality in cognitively able autistic and NT adults [6], less distinct category boundaries should maximize this effect. Finally, participants were introduced to a “yes”– “no” task, rather than a go/no-go task, to avoid an early target–no-target ERP difference due to motor preparation or response inhibition [3].

With respect to behavioral performance, we combined several factors that have been investigated separately in previous studies: (1) category, (2) typicality, and (3) presentation times in (4) both experimental groups. We predict that answers will be faster and discrimination more accurate for animals than for food items, which have less distinct category boundaries. This effect might be enhanced in cognitively able autistic individuals [44]. Regarding typicality, we expect responses to typical items of both categories to be faster and discrimination to be more accurate [20]. Additionally, we predict that longer presentation times will lead to more accurate discrimination and faster responses [6], see also [36]. We expect that there will be no behavioral difference between groups regarding typical or atypical items at short presentation times [66], but at longer presentation times, NT participants will show better discrimination of atypical items compared to cognitively able autistic participants [6].

With respect to the cognitive processes indexed by ERPs, we investigated whether early processing stages of extracting semantic meaning from visual input are influenced by (1) category, (2) typicality, and (3) presentation time and investigated differences between both experimental groups in a comprehensive and qualitative approach. Our first goal was to conceptually replicate the effects in ultra-rapid categorization for NT adults (described by [64], dN150) and extend the effects for cognitively able autistic adults. Additionally, our expectation is that early semantic processing of a category (i.e., dN150) with less distinct category boundaries (i.e., food) might be different from that of a more distinct category (i.e., animal).

In a second step, we explored which aspects of early visual categorization (N1, P2, N2, P2, and P3) are modulated by the cognitive processes under investigation. Specifically, we compared categorization processes across food and animal categories. Since we predict that food items are more difficult to discriminate and the P2 amplitude varies inversely with task difficulty [8], we expected to find lower P2 amplitudes for food compared to animal images for NT and autistic adults. Additionally, the N2 is enhanced by conflict eliciting stimuli, for instance those difficult to classify [71]. Hence, we predict more negative deflections of the N2 component for non-animals compared to animal stimuli [3] for NT and autistic adults.

Moreover, we investigate whether typicality modulates the P2 and P3 components. The anterior P2 component is observed when targets are discriminated based on simple features [35], whereas P3 seems to be elicited during categorization based on arbitrary features [52]. We expect an effect of typicality in the P3 in the autistics, but not in the NT adults, considering that autistic adults processed typical exemplars as efficiently as NT adolescents, but required additional processing for atypical exemplars [26].

Finally, we assessed whether the level of categorization modulates N1 component by including a moderating effect of presentation time, as categorizing visual input on a more detailed level is only possible with longer presentation times. In contrast to the dN150, the N1 component is not observed as a difference wave and is more centrally distribution. In NT adults, the N1 component reflects differences between subordinate and basic level categorization [63]. Hence, we expect an effect of presentation duration in the N1 in NT adults. In contrast, due to the enhanced discrimination abilities of cognitively able autistics, independent of the presentation duration, we do not suspect an effect of presentation duration in the N1 in the cognitively able autistic adults.

Note that these proposed modulations of cognitive processes underlying categorization are not independent, as categorization occurs based on either simple or arbitrary features, i.e., during early or later stages of cognitive processing.

## Method

### Participants

We recruited two groups of participants, NT and cognitively able autistic adults. We choose a male-only sample, since ASD is diagnosed with a 4.3 times higher rate for boys than for girls [39]. All participants reported normal or corrected-to-normal vision and had more than 9 years of formal education. Cognitively able autistic participants scored above 70 points in the verbal subscale of the Wechsler Adult Intelligence Scale and had been diagnosed with ASD (based on DSM-V criteria of the American Psychiatric Association, [2]. Additionally, we used the Asperger’s Syndrome Diagnostic Scale (ASDS;[45]) to confirm the clinical evaluation diagnosis and to characterize the level of autistic traits in this group (see Table 1). The study was conducted according to the Declaration of Helsinki [70] and was approved by the ethical review board of the Faculty of Psychology at the University of Lisbon. All participants gave their written consent after being informed about the procedure and were given the opportunity to ask questions. The data from one cognitively able autistic participant were excluded from further analyses due to low performance (d′ lower than 2 SD below group mean), and the data from one NT adult were excluded due to extensive artifacts (81.7% of all trials had to be removed). The two groups were matched for age, schooling, and general cognitive abilities (assessed with Raven’s progressive matrices) after excluding 4 NT adults with extreme values in age and general cognitive abilities. Thus, the data of 17 NT and 14 cognitively able autistic participants were included in the analysis (for details, see Table 1).

**Table 1:** IQ and demographic information of both groups of male participants, indicated separately for cognitively able autistic and neurotypical (NT) adults

	**Autistic adults**	**NT adults**	***t*-value**	***p*-value**
IQ (mean Raven raw scores)	50.5 (*SD* = 7.7)	53.5 (*SD* = 6.0)	− 0.824	.418
Age (mean in years)	32.5 (*SD* = 8.4)	27.5 (*SD* = 5.4)	1.903	.070
Schooling (mean in years)	14.0 (*SD* = 2.8)	15.8 (*SD* = 2.6)	− 1.834	.078
ASDS (mean raw scores)	103.7 (*SD* = 9.3)			

IQ was measured using Raven’s standard progressive matrices; the average raw scores in a Portuguese NT sample were 41.18 (*SD* = 12.03; [54]). The Asperger’s Syndrome Diagnostic Scale (ASDS; [45]) was used to confirm clinical diagnosis; the standard score (*M* = 100, *SD*** = **15) indicates the probability of autism (Campbell, 2005). *T*-value of pairwise *t*-test for independent groups. *IQ* Intelligence quotient, *SD* Standard deviation

### Materials and procedure

**Fig. 1:**
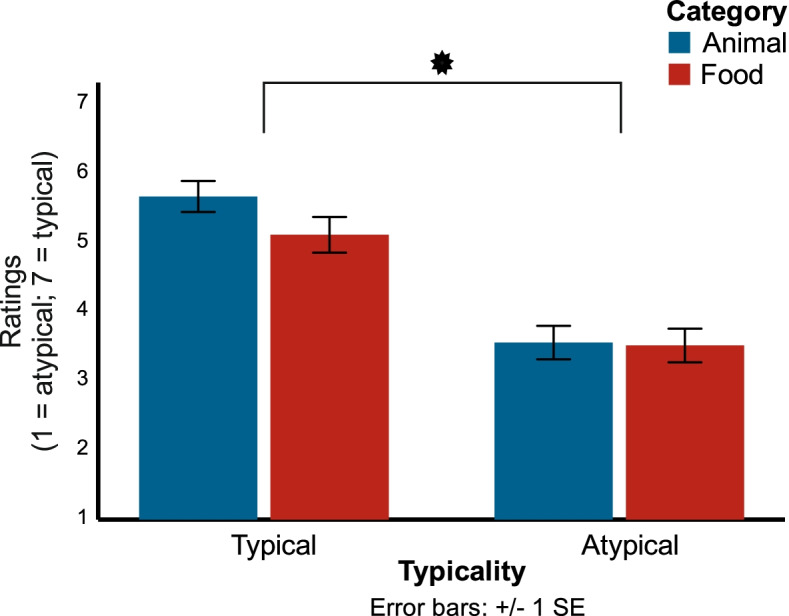
Results of prior rating test. Mean typicality rating of students (*n* = 17) after dividing images in each category (animals in red, food in blue). The asterisk indicates a significant difference between typical and atypical items in both categories

Stimuli were 1600 color photographs; 400 animals and 400 food items were used as targets and 800 objects as non-targets. Targets and non-targets were presented in random order with equal probability (50%). In each trial, one picture of one of these categories was presented. None of these images is generally suspected of producing special emotional arousal. Of course, we cannot exclude such an effect on an individual level. Stimuli were not controlled for color parameters or visual complexity. Throughout the experimental blocks, a black fixation frame at the center of a 10° × 10° visual angle (as described in [67] was visible on a CRT monitor with a refresh rate of 85 Hz and a resolution of 1024 × 768 pixel. Each picture was presented only once in a standardized size (10° × 10° visual angle). In a prior rating test, 17 NT students (mean age = 19.4 years; 2 males) were asked to indicate how well each item represented a given category on a 7-point scale, i.e., “animal” or “food,” respectively. Note that we used an independent sample to rate typicality. The z-transformed means served to define typicality level; values below 0 were used as atypical items and those above 0 as typical items. Based on this definition, the mean original typicality ratings (1–7) were significantly different for both categories (Fig. 1). Hence, 200 images were typical, and 200 images were atypical for each category (i.e., 200 typical animal, 200 atypical animal, 200 typical food, and 200 atypical food images). Stimuli were presented with presentation software (version 18.0, Neurobehavioral Systems). Participants were seated in front of a computer screen, placed at eye level and at an average distance of 40 cm on a table in a dimly lit and shielded room. Participants rested their index fingers on the F and J keys of a QWERTY keyboard and responded by pressing one of the keys. Participants performed a visual categorization task, in which they indicated, for each item, whether or not it belonged to the animal or food category, respectively. Each relevant category was presented in blocks, and the order of these blocks and the response keys (yes, no) were counterbalanced.

**Fig. 2:**
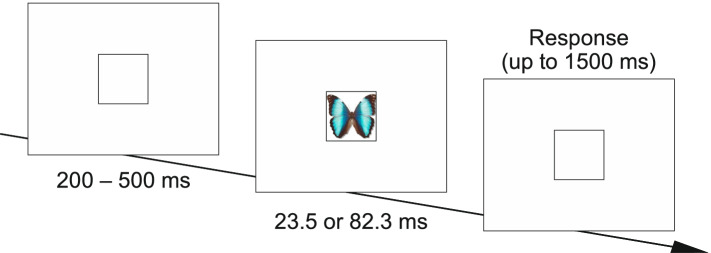
Trial design. For the whole trial and during each block, a fix square was displayed. In each trial, images were presented in the square for either 23.5 or 82.3 ms. Participants could respond until 1500 ms after stimulus offset to a two-option forced choice (“yes”/”no”), answering the question “Did you see an animal image?” Participants were asked to respond as accurately and as fast as possible

Presentation times (short or long) were varied randomly, with equal proportions of targets and non-targets. Stimuli were presented for 2 frames (23.5 ms) in the short condition and for 7 frames (82.3 ms) in the longer presentation duration. Each subsequent stimulus was presented after a random interval between 200 and 500 ms following the response, with a maximum of 1500 ms (displayed in Fig. 2). After every 100 trials, a short break was offered.

### EEG recording

For the EEG recording, we used 64 Ag/AgCl cap-mounted electrodes, plus two placed at the mastoids and four around the eyes, positioned on an extended 10–20 system [25]. The EEG was recorded with the BioSemi EEG-System (BioSemi B.V., Amsterdam, Netherlands). All electrodes were recorded with an electrode offset within a 40-μV range. The electrode offset is generated at the junction of the skin and electrolyte solution under the electrodes. It is a by-product of the direct current potentials and results in a voltage at the amplifier input (Jones, 2015). We used the electrodes around the eyes (above and below the right eye and beside the right and left eye) to record eye movements. The ground electrode was placed with the common mode sense (CMS) active electrode and the driven right leg (DRL) passive electrode at the electrode positions PO1 and PO2, respectively, in the 10–20 system. The CMS is also used as online reference. The sampling frequency was 2048 Hz. EEG signal was filtered online with a 0.16 Hz high-pass filter and a 100 Hz low-pass filter.

### EEG data processing

We used the spherical spline method [50] for interpolation of electrodes with many artifacts, since this method makes no assumption about the conductivity of the head tissues [9],on average in NT adults: 1.9 electrodes; in cognitively able autistic adults: 2.6 electrodes, ranging for both groups between 0 and 8 interpolated electrodes). The signal was re-referenced offline to the average of all cap-mounted electrodes using BrainVision Analyzer 2.1 (Brain Products GmbH, Gilching, Germany). The choice of reference depends on a variety of factors, including the number of electrodes, location of electrodes, cognitive task, analyses to be performed, and brain regions to be investigated (see [9]. Since most of the relevant portions of ERPs in cognitive neuroscience consist of frequencies between 0.01 and 30 Hz [35], the EEG signal was filtered using a zero-phase shift Butterworth filter (most common used filter [9], with a high cutoff at 30 Hz at 48 dB/oct. We corrected for eye movement artifacts by using an independent component analysis (ICA) with the infomax-restricted algorithm [28], where possible. For the ICA, we selected a 100 s interval from the 16th block of the experiment as a training data set for computing the unmixing matrix. ICA components were automatically identified by picking up blinks and saccades, as evidenced by their characteristic shape and maximum at frontal sites. After removing these components, the EEG was reconstructed. Two cognitively able autistic participants did not blink during most of the blocks,thus, a suitable amount of data for the ICA was not available [23]. Therefore, we manually deleted all blinks for these participants, which amounted to 38 and 49 blinks, resulting in 1.38 % and 2.21 % deleted trials, respectively. EEG segments were based on a time window of 200 ms before and 800 ms after stimulus onset. Artifacts were removed automatically when (1) the amplitude difference between two sample points exceeds 50 μV, (2) the amplitude difference was more than 150 μV in an interval of 100 ms, or (3) a low amplitude of 0.5 µV occurred in a 100 ms interval [9]. Due to the artifact rejection, on average, 2.48 % of all trials had to be removed in the NT group and 5.07 % in the cognitively able autistic group. On average, there were 114 (range: 49–197) trials left per condition per participant for the NT participants and 109.9 (range: 33–196) for the cognitively able autistic participants (for more details, see Table 2).

**Table 2:** Trials per condition. Mean number of trials and range, per condition, used in the ERP analysis, calculated for cognitively able autistic adults and neurotypical (NT) adults separately

Condition	NT adults (mean trials)	NT adults (range)	Autistic adults (mean trials)	Autistic adults (range)
Non- food, 82.3 ms presentation time	173.5	128–196	170.2	136–195
Non- food, 23.5 ms presentation time	169.2	120–192	162.8	131–191
Non- animal, 82.3 ms presentation time	174.4	143–197	168.9	122–195
Non- animal, 23.5 ms presentation time	171.7	128–197	164.1	126–196
Food, typical, 82.3 ms presentation time	89.3	67–102	87.1	65–109
Food, typical, 23.5 ms presentation time	89.6	57–113	82.4	53–99
Food, atypical, 82.3 ms presentation time	81.3	54–101	78.4	62–90
Food, atypical, 23.5 ms presentation time	70.2	49–90	68.4	33–91
Animal, typical, 82.3 ms presentation time	99.7	75–122	95.9	64–112
Animal, typical, 23.5 ms presentation time	98.8	82–114	95.6	66–116
Animal, atypical, 82.3 ms presentation time	76.3	63–86	73.4	47–89
Animal, atypical, 23.5 ms presentation time	74.4	56–92	71.9	52–83

Note that non-food and no-animal conditions were only used for the dN150 analysis

### Analysis of behavioral data

Category discrimination (d′) and reaction times (RTs) were analyzed. For both, responses faster than 200 ms were excluded, resulting in the exclusion of 2.82 % of all trials in the NT group and 2.64 % in the cognitively able autistic group. Statistical analyses were restricted to responses to the target category. RT analyses were based on correct answers only; hence, an average of 10.29 % of all trials had to be excluded in the NT group and 9.64 % in the cognitively able autistic group due to incorrect responses.

Category discrimination was analyzed using d prime (d′). D′ was calculated by using the z score of the probability of hits minus the z score of the probability of false alarms. In the absence of false alarms or misses, we applied the formula by Macmillan and Creelman [37]. A d′ of 0 indicates no discrimination, whereas a d′ of 4 indicates nearly perfect performance [38]. For both mean d′ and mean RTs, we used a repeated measure analysis of variance (ANOVA) with category (animal vs. food), typicality (typical vs. atypical), and presentation time (23.5 vs. 82.3 ms) as within subject factors and group (NT vs. autistic participants) as between subject factor. For the sake of brevity and to ease readability, we report only those effects and interactions with *p*-values below the conventional significance value of .05; all remaining analyses are not listed in the result section. All recorded *p*-values were Greenhouse–Geisser corrected, when needed [22].

### Analysis of EEG data

A baseline correction was applied to the segmented signal, using the time window of 200 ms before stimulus onset (as recommend by [35]). The signal was averaged per condition and participant. For the first part of the EEG analysis, we compared the averaged signal of correct responses to target and distractor images to replicate the results by Thorpe et al. [64]. We also followed the statistical approach used in that study, i.e., we tested when the onset of the differential activity (targets–non-targets), dN150, diverges from 0 (15 consecutive *t*-test values below *p* < .01; [57] as used by [64]). Since Thorpe et al. [64] recorded with a sampling rate of 1000 Hz, we downsampled our EEG signal to this value. Similar to Thorpe et al. [64, 67], we grouped the signal of seven electrodes (Fp1, Fp2, F3, F4, F7, F8, and Fz) for a frontal region of interest (ROI) and six electrodes (O1, O2, Oz, PO7, PO8, and POz) for an occipital ROI for the short (23.5 ms) and the long (82.3 ms) presentation duration with Python 3.6.5. To calculate the peak latency of the differential activity, we selected individual peaks occurring between 150 and 200 ms, for each both ROIs for each presentation durations, with BrainVision Analyzer. To assess whether the peak latencies differed between conditions in each group, we used a repeated measure ANOVA with two variables of two levels, ROI (frontal vs. occipital), category (animal vs. food), and presentation duration (23.5 vs. 82.3 ms) with SPSS 26. To increase readability, we report only main or interaction effects involving the factors category or presentation duration.

In the second part of the EEG analysis, we focused on the N1, P2, N2, and P3 components based on the literature. For these analyses, we only used target stimuli to which participants responded correctly with a minimum RT of 200 ms, in line with the criteria used for behavioral data. These effects were evaluated at pre-specified ROIs according to the literature. The N1 component peaks at around 140 ms after stimulus onset; therefore, we chose a time window between 120 and 170 ms (similar to [52]) to calculate the peak latency and mean amplitude. The ROI for this analysis comprised the average signal of F3, F4, Fz, C3, C4, and Cz (similar to [3]). We calculated the peak latency and mean amplitude for the anterior P2 between 180 and 240 ms (similar to [8]), at an anterior ROI including F1, F2, Fz, FC1, FC2, FC5, FC6, FCz, C3, C4, C5, C6, and Cz [8]. For the anterior N2, we used the time window between 240 and 300 ms (similar to [8]) with a ROI containing Fpz, Fp1, Fp2, AFz, AF3, AF4, Fz, F1, and F2 [40]. The P3 was analyzed between 300 and 500 ms after stimulus onset [52] at a central-parietal ROI (averaged P1, P2, Pz, CP1, CP2, CPz, C1, C2, and Cz, similar to [52]). To assess the peak latency, we used local maxima/minima to identify the peaks in the pre-specified ROI and time window of each ERP component for each participant. This procedure identifies the latency corresponding to the largest/smallest amplitude value in the specified time window and ROI.

For mean amplitudes, an omnibus ANOVA with the factors Group, ROI, Category, Typicality, and Presentation Time for the entire time window of interest (150–500 ms) revealed a significant 5-way interaction (*p* < .05). A corresponding omnibus ANOVA with the factors Group, Component, Category, Typicality, and Presentation Time for peak latencies revealed another significant 5-way interaction (*p*** < **.05), demonstrating the complex interplay of all the factors under investigation for both types of dependent variables. These interaction effects were followed up by performing hierarchical ERP analyses by group, since this approach is required to account for the specifics of event-related potentials from a theoretical perspective; event-related potentials assess the average EEG activity across many trials in order to enhance systematic activity associated specifically with cognitive processing related to a stimulus. This approach effectively reduces the impact of unsystematic fluctuations in the ongoing EEG (i.e., improving the signal-to-noise ratio). However, this approach does not control for factors unrelated to cognitive activity that do not vary across trials (i.e., morphological differences between individuals like skull thickness or myelination) but still can have considerable influence on amplitude differences as measured on the skull. Therefore, comparing microvolt differences in amplitude between individuals does not allow to make inferences about the underlying cognitive processes (c.f. [41, 65]). Moreover, numerous neuroanatomical differences have been described for autistic as compared to NT individuals (for review, see [1]), for instance, differences in total brain volume, regional gray — white matter differences, differences in stacking of neuronal cell bodies (particularly in layers III and V of the neocortex), and differences in the density of the cerebellum and the size of the amygdala [1]. Additionally, anomalies have been found in the biosynthesis and transmission [4, 15–17, 46–48, 53, 60, 72] as well as expression [10, 56] of the excitatory neurotransmitter glutamate and the inhibitory neurotransmitter gamma-aminobutyric acid in autistic individuals. As these differences in morphology are unlikely to be randomly distributed, EEG waveforms vary systematically in morphology across participant groups, and even with the poor spatial resolution of the surface EEG, these effects are not minimized. Hence, ERP analyses were performed for each group separately, as statistical interactions between groups of participants may reflect anatomical differences rather than different cognitive processes employed in each group.

For each component, we used a repeated measure ANOVA with the following factors: Category (animal vs. food), Typicality (typical vs. atypical), and Presentation Time (23.5 vs. 82.3 ms) for each group. Similar to behavioral analyses, we report effects with *p*-values below the conventional significance criterion of .05. This also includes significant interaction effects that did not reveal any significant differences between conditions in Bonferroni-corrected post hoc comparisons. In addition, we report all results of special interest (i.e., those explicitly based on the hypotheses or effects that reach statistical significance in one group, but not in the other.) We illustrate the results of these ERP analyses in the figures, focusing on significant main or interaction effects. After the data were extracted for statistical analyses, a 20 Hz high cutoff filter (at 48 dB/oct) was applied to increase readability of the figures. Figures were created in the BrainVision Analyzer and were edited with CorelDraw X7 (Corel GmbH, München, Germany). For the ERP figures, we edited the following aspects in CorelDraw X7: color and thickness of the ERP waves, thickness of the x- and y-axis, font size of the values on the x- and y-axis, added either a gray square to indicate the time window or arrows to indicate the peak an analysis was performed on, adding asterisk to indicate significant differences, added legends, and crop the width of the whole figure at appropriate millisecond to represent the ERP results. For the topographical maps, we added black dots for the electrodes included in the ROI and increased the numbers on the legend in CorelDraw X7.

## Results

### Results of behavioral data

For D′, we found a main effect of Category, *F*(1.29) = 43.74, *p* < .001, *η*_p_^2^ = .60; Typicality, *F*(1.29) = 19.66, *p* < .001, *η*_p_^2^ = .40; and Presentation Time, *F*(1.29) = 96.33, *p* < .001, *η*_p_^2^ = .76. Participants were better in detecting animal than food images, with a mean d′ of 3.23 (*SE* = 0.14) and 2.72 (*SE* = 0.13), respectively. Discrimination performance was higher for typical items (*M* = 3.05, *SE* = 0.14) than for atypical items (*M* = 2.91, *SE* = 0.13). Performance was lower for short presentation times (*M* = 2.73, *SE* = 0.13) than when presentation time was long (*M* = 3.22, *SE* = 0.13). In line with our hypothesis, we found an interaction effect between Category and Typicality, *F*(1.29) = 22.15, *p* < .001, *η*_p_^2^ = .43, with differences between typical (*M* = 2.86, *SE* = 0.15) and atypical images (*M* = 2.58, *SE* = 0.12; *p* < .001) occurring only in the food category, and not in the animal category (*p* = .99). We also found an interaction between Category and Presentation Time (*F*(1.29) = 7.46, *p* < .05, *η*_p_^2^ = .21), with a significant difference between the two presentation times (*p* < .001) for both animal and food stimuli. This difference was larger for the food (*M*_Difference_ = 0.58) than the animal (*M*_Difference_ = 0.37) category.

For RT, as for D′, we found a main effect of Category, *F*(1.29) = 38.65, *p* < .001, *η*_p_^2^ = .57; Typicality, *F*(1.29) = 16.21, *p* < .001, *η*_p_^2^ = .35; and Presentation Time, *F*(1.29) = 659.43, *p* < .001, *η*_p_^2^ = .95. Participants were faster for animal than for food images, with mean reaction times of 453 ms (*SE* = 16 ms) and 514 ms (*SE* = 17 ms), respectively. Additionally, responses to typical items were faster (*M* = 479 ms, *SE* = 15 ms) than responses to atypical items (*M* = 487 ms, *SE* = 16 ms). Responses were considerably slower for short (*M* = 523 ms, *SE* = 16 ms) compared to long presentation time (*M* = 444 ms, *SE* = 16 ms). Moreover, we found an interaction between Category and Presentation Time (*F*(1.29) = 10.88, *p* < .01, *η*_p_^2^ = .27), with the shortest RTs observed when participants responded to animal images presented for 82.3 ms (*M* = 417 ms, *SE* = 16 ms) and the longest RTs observed when food images were presented for 23.5 ms (*M* = 558 ms, *SE* = 18 ms). As described for D′, for both animal and food stimuli, we observed differences between presentation times (*p* < .001), with larger differences found for food stimuli (*M*_Difference_ = 87 ms) than for stimuli from the animal category (*M*_Difference_ = 71 ms).

Taken together, behavioral data indicate faster responses to, and more accurate discrimination of typical than atypical items, and for the animal compared to the food category. We did not observe differences in behavioral performance between the NT and cognitively able autistic group. Consequently, ERP data — which are analyzed for correct responses only (see Table 2) — have a comparable signal-to-noise ratio in both groups. This contributes to the reliability of ERP waveforms in both groups, simultaneously ruling out a potential confound of lower performance for the clinical population in particular.

### Results of EEG data for the NT adults

**Fig. 3:**
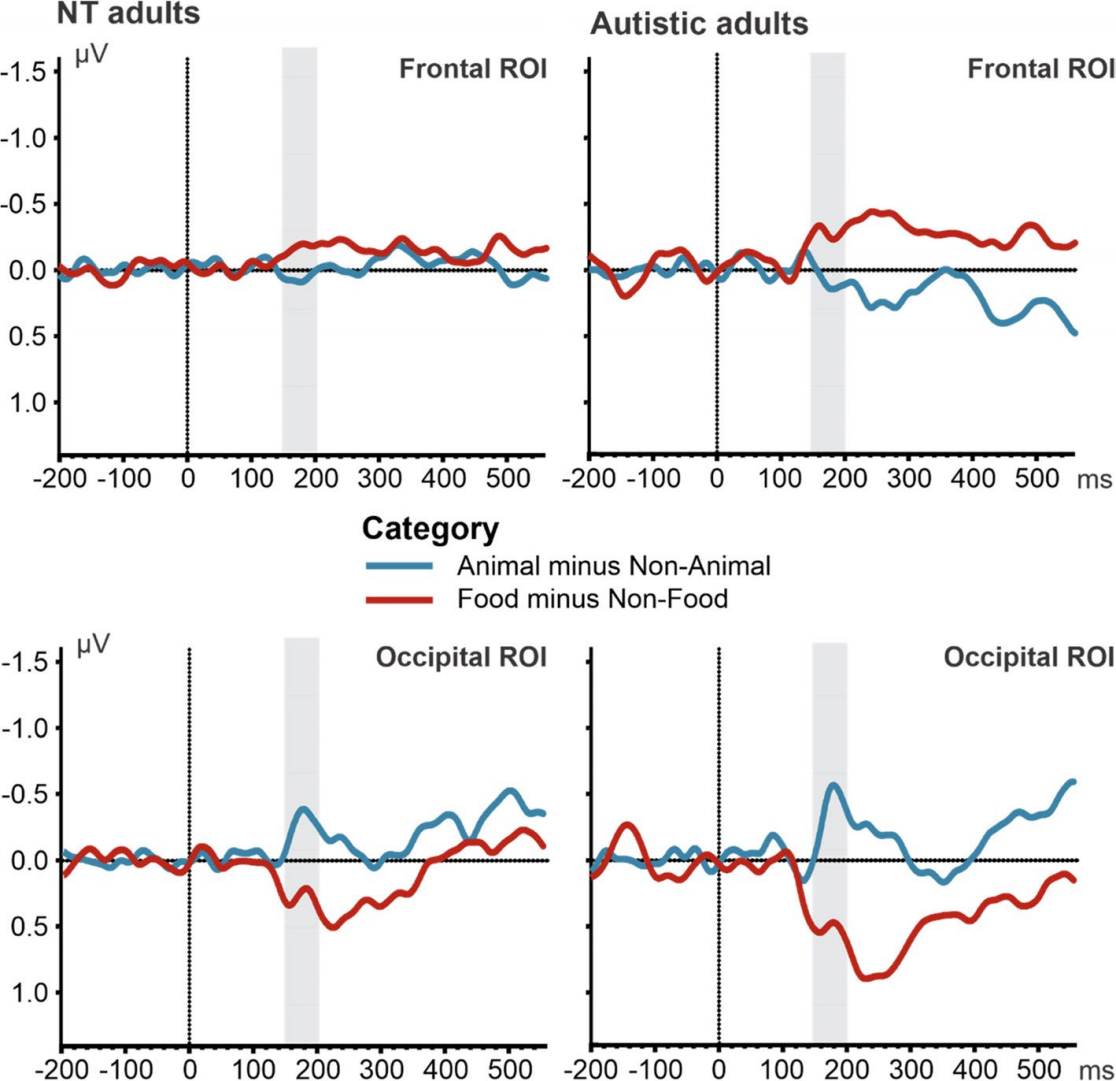
Difference (dN150) between target and nontarget stimuli in the frontal (including electrodes site Fp1, Fp2, F3, F4, F7, F8, and Fz) and occipital ROI (including electrode sites O1, O2, Oz, PO7, PO8, and POz) for cognitively able autistic and NT adults. The time window used for the peak detection is illustrated by the gray bar over the x-axis (i.e., ms). A repeated measure ANOVA was performed for each group with the factors: ROI (frontal vs. occipital) and category (animal vs. food). We observed a difference between the difference of animals minus non-animals and food minus non-food in the frontal and occipital ROI in both the NT and the cognitively able autistic adults

We calculated the difference wave between the waveforms corresponding to target and non-target stimuli and observed reliable divergences from 0 (defined as significant differences in 15 consecutive *t*-tests, cf. [57] as used by [64]) in the frontal ROI in both groups. For NT adults, the divergence from 0 can only be observed in the following condition: food and non-food stimuli started to diverge 192 ms following a short stimulus presentation (Fig. 3). In the other conditions, no reliable divergence was observed between 150 and 200 ms.

In addition, we analyzed peak amplitudes and latencies of the difference between target and non-target stimuli in the time window 150–200 ms. The most obvious feature of the dN150 at visual inspection was that the difference waves for targets minus non-targets differed in polarity depending on category and ROI. Hence, peak detection identified maximal distance from zero, i.e., positive peaks in the frontal ROI for the difference between animal and non-animal images and negative peaks for food/no-food images, and the reverse in the occipital ROI. In NT adults, we observed a main effect of category, *F*(1.16) = 7.93, *p* < .05, *η*_p_^2^ = .33; a larger peak amplitude was observed in the animal (*M* =  − 0.16 μV, *SE* = 0.07 μV) compared to the food category (*M* = 0.05 μV, *SE* = 0.05 μV). Lastly, we observed an interaction effect between ROI and category, *F*(1.16) = 14.37, *p* < .05, *η*_p_^2^ = .47, with a difference between both categories in the frontal (*p* < .05) and the occipital ROI (*p* < .005) with a reversed pattern of amplitudes. In the frontal ROI, the magnitude of the peak amplitude was larger for the animal (*M* = 0.51 μV, *SE* = 1.00 μV) than the food category (*M* = 0.27 μV, *SE* = 0.08 μV). Similarly, in the occipital ROI, the magnitude of the peak amplitude was larger for the animal (*M* =  − 0.83 μV, *SE* = 0.15 μV) than the food category (*M* =  − 0.18 μV, *SE* = 0.12 μV). Note that these values refer to the magnitude of the difference waves (i.e., polarity depends on how the difference is computed; see Table 3). Taken together, the effect of category was observed in the ROIs with a larger peak amplitude for animals than for food. Importantly, the duration of the presentation did not affect amplitude or latency of the difference waveforms.

**Table 3:** Mean peak amplitudes and latencies of the divergence between target and nontarget stimuli in the 150 and 200 ms time window

	**NT adults**				**Autistic adults**			
	**Animal 23.5 ms**	**Animal 82.3 ms**	**Food 23.5 ms**	**Food 82.3 ms**	**Animal 23.5 ms**	**Animal 82.3 ms**	**Food 23.5 ms**	**Food 82.3 ms**
**Peak amplitude in μV**								
Frontal ROI	0.69 (0.17)	0.33 (0.16)	0.43 (0.13)	0.10 (0.1)	0.45 (0.17)	0.66 (0.26)	0.19 (0.2)	0.11 (0.27)
Occipital ROI	− 0.93 (0.2)	− 0.72 (0.25)	− 0.35 (0.16)	− 0.01 (0.11)	− 0.72 (0.15)	− 1.1 (0.22)	− 0.84 (0.26)	0.01 (0.29)
**Peak latency in ms**								
Frontal ROI	179 (4)	173 (3)	173 (4)	172 (4)	177 (3)	176 (2)	171 (3)	176 (4)
Occipital ROI	176 (4)	179 (3)	181 (3)	178 (3)	182 (3)	178 (2)	174 (4)	182 (4)

Mean values were calculated for cognitively able autistic and neurotypical (NT) adults separately (standard error in brackets). Values of peak amplitude are in μV, whereas peak latencies are in ms. The frontal ROI included the following electrode sites: Fp1, Fp2, F3, F4, F7, F8, and Fz. The occipital ROI included the following electrode sites: O1, O2, Oz, PO7, PO8, and POz. In the animal task, we compared animal and no-animal images; in the food task, we compared food and no-food images

**Fig. 4:**
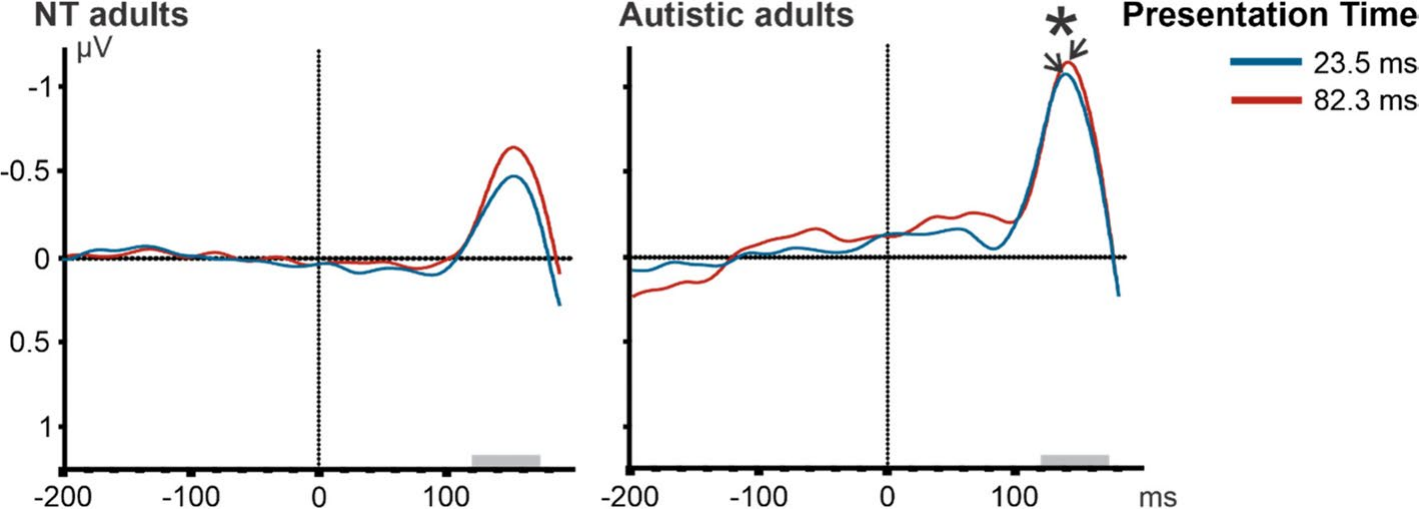
ERP waveforms illustrating N1 effects for cognitively able autistic and NT adults for target stimuli in a frontal ROI comprising the following electrode sites: F3, F4, Fz, C3, C4, and Cz. The time window used for the peak detection is illustrated by the gray bar over the x-axis (i.e., ms). A repeated measure ANOVA was performed for each group with the factors: category (animal vs. food), typicality (typical vs. atypical), and presentation time (23.5 vs. 82.3 ms). In cognitively able autistic adults, a peak latency (indicated by arrow) difference was observed between short (blue) vs. long (red) presentation time, as indicated by the asterisk

**Fig. 5:**
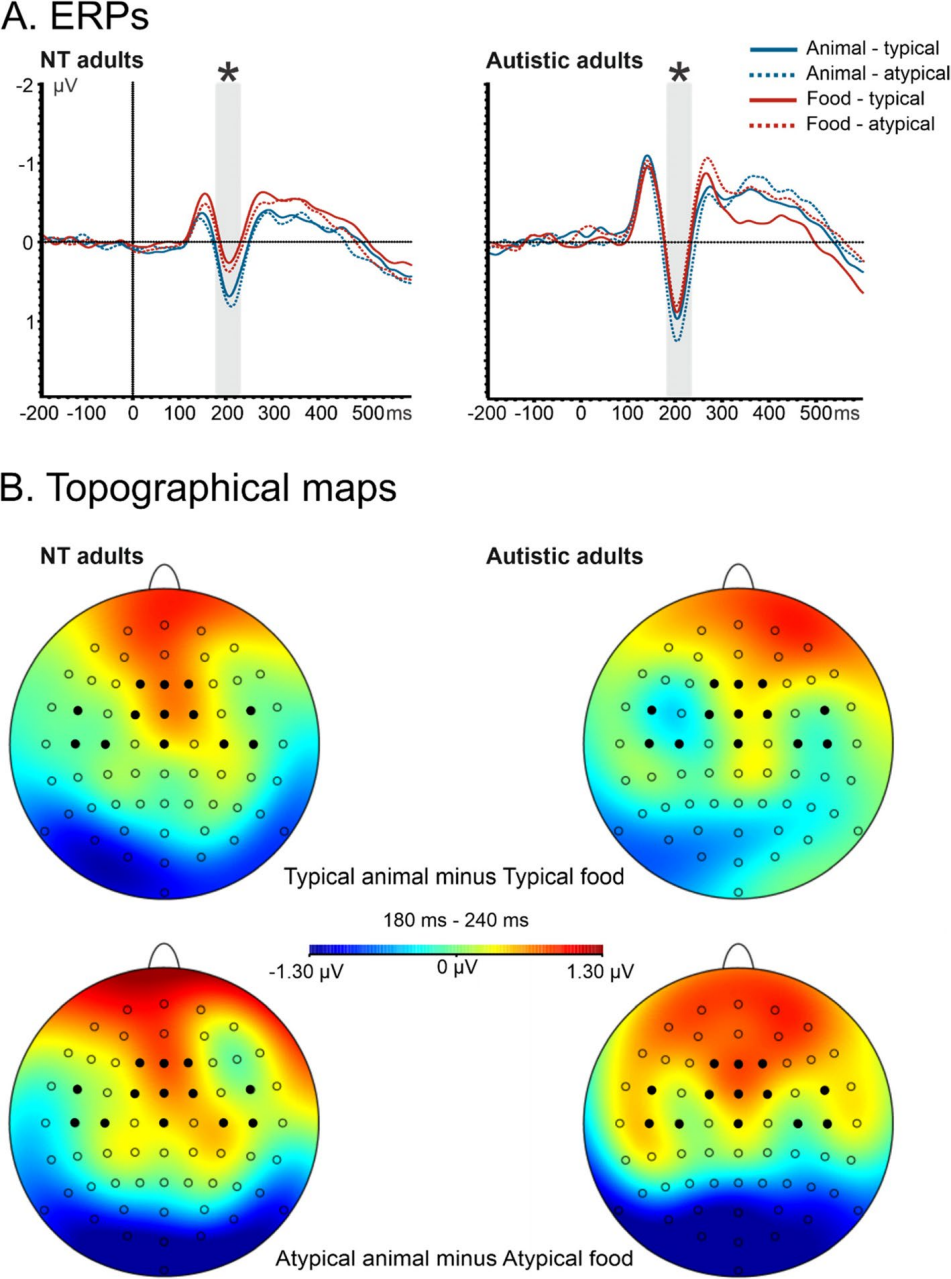
ERP waveforms illustrating P2 effects for cognitively able autistic and NT adults for target stimuli in a ROI comprising the following electrode sites: F1, F2, Fz, FC1, FC2, FC5, FC6, FCz, C3, C4, C5, C6, and Cz. A repeated measure ANOVA was performed for each group with the factors as follows: category (animal vs. food), typicality (typical vs. atypical), and presentation time (23.5 vs. 82.3 ms). The results reflect influences of category (food vs. animal) and typicality (typical vs. atypical). **A** ERPs. The time window used for the mean activity is illustrated by the gray bar over the x-axis (i.e., ms). In both groups, we observed effects of typicality and category, as indicated by the asterisk. In NT adults, we observed differences between animal (blue) vs. food (red) and typical (continuous) vs. atypical (dotted) images.** B** Topographical maps. Electrode sites are marked with circles, filled circle indicates the ROI for this analysis. In cognitively able autistic adults, we observed a difference between atypical animal vs. atypical food images, but not between typical animal vs. typical food images

**Fig. 6:**
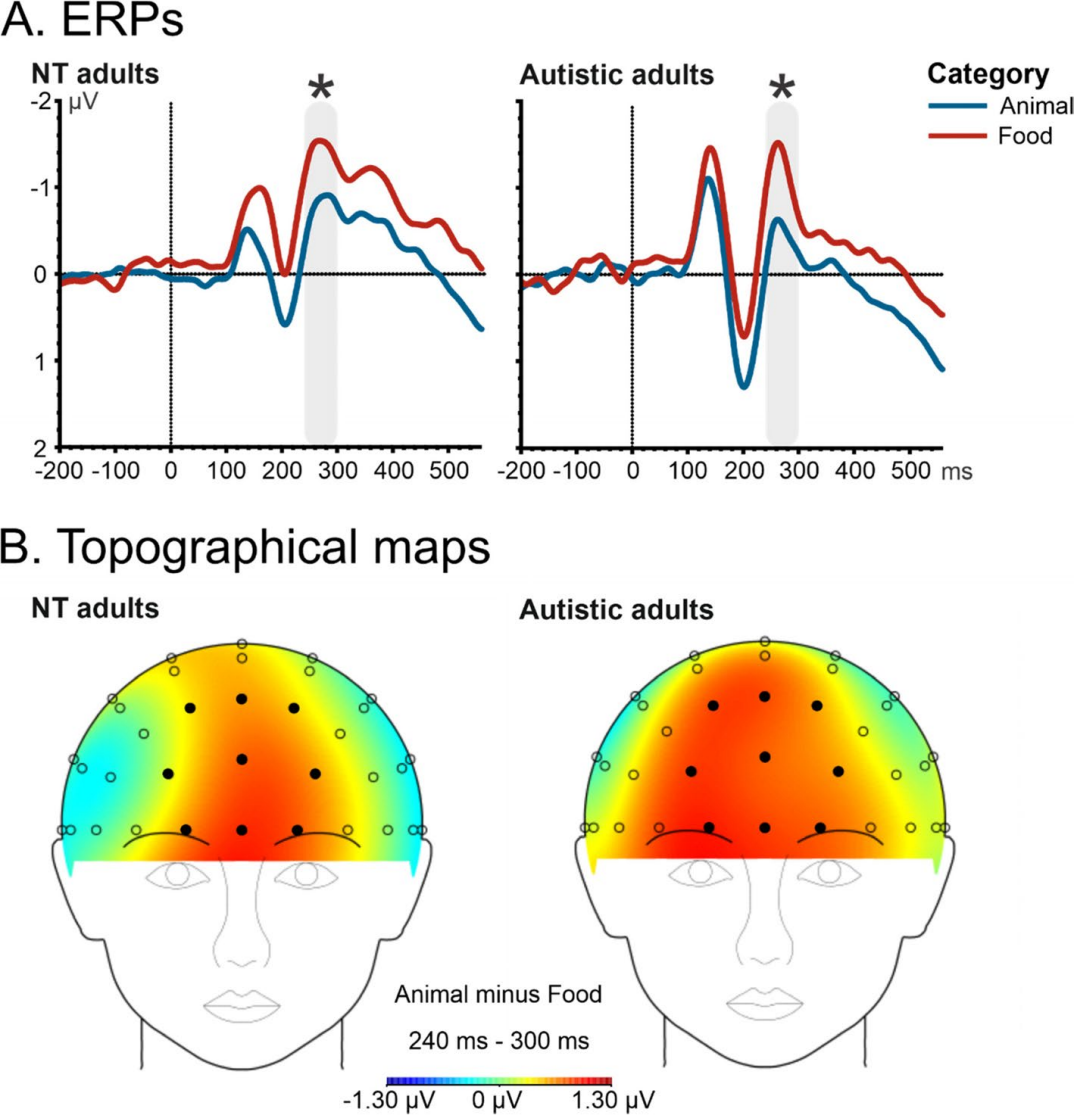
ERP waveforms illustrating N2 effects for cognitively able autistic and NT adults for target stimuli in a ROI using the following electrode sites: Fpz, Fp1, Fp2, AFz, AF3, AF4, Fz, F1, and F2. A repeated measure ANOVA was performed for each group with the factors: category (animal vs. food), typicality (typical vs. atypical), and presentation time (23.5 vs. 82.3 ms). In both groups, we observed differences between the animal vs. the food category. **A** ERPs. The time window used for the mean activity is illustrated by the gray bar over the x-axis (i.e., ms). In both groups, we observed differences between animal (blue) vs. food (red) images, as indicated by the asterisk. **B** Topographical maps. Electrode sites are marked with circles; filled circle indicates the ROI for this analysis

**Fig. 7:**
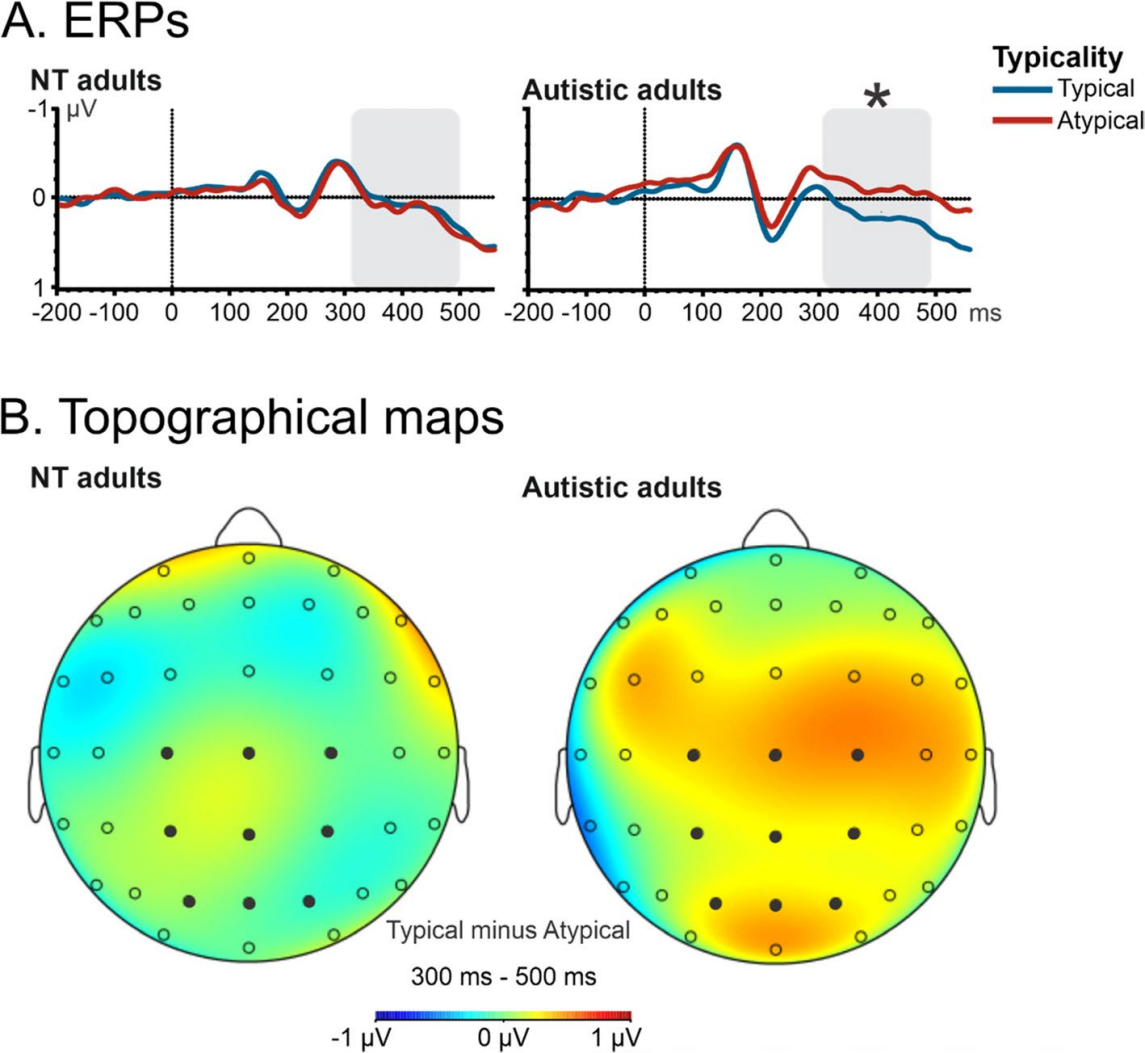
ERP waveforms illustrating P3 effects for cognitively able autistic and NT adults for target stimuli in a ROI using the following electrode sites: P1, P2, Pz, CP1, CP2, CPz, C1, C2, and Cz. A repeated measure ANOVA was performed for each group with the factors as follows: category (animal vs. food), typicality (typical vs. atypical), and presentation time (23.5 vs. 82.3 ms). Only for cognitively able autistic adults reliable differences between typical vs. atypical images were observed. **A** ERPs. The time window used for the mean activity is illustrated by the grey bar over the x-axis (i.e. ms). In cognitively able autistic adults, we observed differences between typical (blue) vs. atypical (red) images, as indicated by the asterisk. **B** Topographical maps. Electrode sites are marked with circles; filled circle indicates the ROI for this analysis

Complementing the classic approach of Thorpe and colleagues, we also analyzed mean amplitudes and mean peak latencies for the N1, P2, N2, and P3 component. A repeated measure ANOVA was performed with the factors: Category (animal vs. food), Typicality (typical vs. atypical), and Presentation Time (23.5 vs. 82.3 ms), for each component. With respect to the N1 component (120 − 70 ms), we did not observe an effect in NT adults (Fig. 4). For the mean amplitude of the anterior P2 component (180–240 ms; Fig. 5), the ANOVA showed a main effect of Category, *F*(1.16) = 16.41, *p* < .001, *η*_p_^2^ = .51, with a more positive value for animal images (*M* = 0.44 μV, *SE* = 0.14 μV) than food images (*M* = 0.08 μV, *SE* = 0.15 μV). We also found a main effect of Typicality, *F*(1.16) = 9.72, *p* < .001, *η*_p_^2^ = .38, with a smaller amplitude for typical (*M* = 0.19 μV, *SE* = 0.15 μV) than for atypical images (*M* = 0.33 μV, *SE* = 0.13 μV). For the anterior N2 component (240–300 ms; Fig. 6), in NT adults, mean amplitude was influenced by Category, *F*(1.16) = 5.34, *p* < .05, *η*_p_^2^ = .25, and Presentation Time, *F*(1.16) = 9.97, *p* < .001, *η*_p_^2^ = .38. We observed a smaller N2 in the animal task (*M* =  − 0.7 μV, *SE* = 0.36 μV) than in the food task (*M* =  − 1.29 μV, *SE* = 0.5 μV), in addition to a smaller N2 amplitude for the short (*M* =  − 0.72 μV, *SE* = 0.37 μV) than the long (*M* =  − 1.26 μV, *SE* = 0.55 μV) presentation time. For P3 (300–500 ms) component, we observed no significant effects (Fig. 7).

### Results of EEG data for autistic adults

For the cognitively able autistic adults, the divergence from 0 can only be observed in one condition; animal and non-animal stimuli started to diverge 171 ms following a long presentation duration (Fig. 3). In the other conditions, no reliable divergence was observed between 150 and 200 ms.

In addition, we analyzed peak amplitudes and latencies of the difference between target and non-target stimuli in the time window 150–200 ms. In cognitively able autistic adults, we observed a main effect of Category, *F*(1.16) = 5.55, *p* < .05, *η*_p_^2^ = .30, with a larger peak amplitude in the animal (*M* =  − 0.18 μV, *SE* = 0.05 μV) compared to the food category (*M* = 0.06 μV, *SE* = 0.07 μV). Lastly, we observed an interaction effect between ROI and Category, *F*(1.13) = 11.02, *p* < .01, *η*_p_^2^ = .46, as the difference between both categories in the frontal (*p* < .05) and the occipital electrode site (*p* < .01) showed a reversed polarity. In the frontal ROI, the peak amplitude was larger for the animal (*M* = 0.56 μV, *SE* = 0.07 μV) than the food category (*M* = 0.15 μV, *SE* = 0.17 μV). Similarly, in the occipital ROI, the magnitude of the peak amplitude was larger for the animal (*M* =  − 0.91 μV, *SE* = 0.15 μV) than the food category (*M* =  − 0.04 μV, *SE* = 0.22 μV). However, as noted above, this analysis detects differences between both conditions; hence, the numbers reflect relative values only. Taken together, the category effect was observed in the ROIs with a larger peak amplitude for animals than for food. Importantly, the duration of the presentation did not affect amplitude or latency of the difference waveforms.

Complementing the classic approach of Thorpe and colleagues, we also analyzed mean amplitudes and mean peak latencies for the N1, P2, N2, and P3 component. With respect to peak latencies of the N1 component (120–170 ms), we observed a main effect of Presentation Time in cognitively able autistic individuals, *F*(1.13) = 9.08, *p* < .01, *η*_p_^2^ = .41, with an earlier peak for the short (*M* = 146 ms, *SE* = 3 ms) compared to the long presentation time (*M* = 149 ms, *SE* = 3 ms; Fig. 4). For the mean amplitude of the anterior P2 component (180–240 ms) in the cognitively able autistic adults, we also observed a larger (i.e., more positive) P2 for animal images (*M* = 0.74 μV, *SE* = 0.33 μV) than for food images (*M* = 0.43 μV, *SE* = 0.34 μV). This main effect of Category, *F*(1.13) = 5.78, *p* < 0.05, *η*_p_^2^ = 0.31 was modulated by an interaction effect between category and typicality, *F*(1.13) = 16.07, *p* < .001, *η*_p_^2^ = 0.55, with no effect of category in typical images (*p* = .99) but a significant difference between atypical animal images (*M* = 0.91 μV, *SE* = 0.33 μV) and atypical food images (atypical: *M* = 0.38 μV, *SE* = 0.34 μV; *p* < 0.05; Fig. 5). For the anterior N2 component (240–300 ms), we observed a main effect of Category, *F*(1.13) = 6.36, *p* < .05, *η*_p_^2^ = .33, with a smaller mean N2 amplitude for animal (*M* =  − 0.43 μV, *SE* = 0.48 μV) than for food (*M* =  − 1.2 μV, *SE* = 0.41 μV) images (Fig. 6). For P3 (300–500 ms) amplitudes, we observed a significant effect in typicality, *F*(1.13) = 4.71, *p* < .05, *η*_p_^2^ = .27. We observed a larger P3 in typical (*M* = 0.17 μV, *SE* = 0.35 μV) compared to atypical (*M* =  − 0.13 μV, *SE* = 0.36 μV) images (Fig. 7).

To summarize, we found evidence that all three factors under investigation — category, typicality, and presentation time — modulated specific aspects of semantic categorization (see Table 4). In both groups, we observed similar effects of category in the mean amplitude of P2 and N2, specifically smaller P2 and larger N2 amplitudes for food stimuli compared to animal stimuli. In contrast, the effect of typicality manifested as smaller P2 amplitudes for typical images in NT adults and larger P3 amplitudes for typical images in cognitively able autistic adults. Finally, presentation times affected the amplitude of N2 in NT adults and the latency of N1 in cognitively able autistic adults; we observed a larger mean N2 amplitude for longer presentation times in NT adults and a shorter N1 peak latency for short presentation times in cognitively able autistic adults.

**Table 4:** Summary of statistically significant (*p* < .05) ERP results

	**Theoretical meaning of the component**	**NT adults**		**Autistic adults**	
		**Mean amplitude in μV**	**Peak latency in ms**	**Mean amplitude in μV**	**Peak latency in ms**
**N1 (120–170 ms)**	Differences between subordinate and basic level categorization [63]				Presentation time
**P2 (180–240 ms)**	Inversely correlation with task difficulty [8] Target discrimination based on simple features [35]	Category Typicality		Category Category × Typicality	
**N2 (240–300 ms)**	N2 is enhanced by those difficult to classify [71]	Category Presentation Time		Category	
**P3 (300–500 ms)**	Categorization based on arbitrary features [52]			Typicality	

Analyses were done separately for each group (cognitively able autistic and NT adults), including the factors category (animal vs. food), typicality (typical vs. atypical), and presentation time (23.5 vs 82.3 ms)

## Discussion

In the present study, we assessed how category, typicality, and presentation time (23.5 vs. 82.3 ms) affect behavioral and neural responses in ultra-rapid categorization. Specifically, we compared NT and cognitively able autistic adults during semantic categorization of animal vs. non-animal (discrete category boundaries) and food vs. non-food (fuzzy category boundaries) stimuli. We (1) conceptually replicated the ultra-rapid design introduced by Thorpe et al. ([64],dN150), (2) evaluated potential effects of a less distinct category (animal vs food stimuli), and (3) assessed whether typicality influences simple (anterior P2) or abstract (P3) feature categorization. Finally, we (4) used the N1 and N2 component as references to assess the association between presentation time and hierarchical level of categorization. Both discrimination ability and RTs confirm that it is more difficult to categorize food compared to animal stimuli, as well as atypical compared to typical images. Longer stimulus presentation times enhanced behavioral performance, particularly for the food category with less specific category boundaries. Notably, we did not observe performance differences between cognitively able autistic and NT adults. In a rapid-serial visual presentation paradigm, however, Carmo et al. [6] reported group differences in semantic categorization, with cognitively able autistic participants unable to detect atypical items even with the longest presentation times. Simplified response requirements and overall longer presentation times in the present paradigm made the task easier for all participants, eliminating a potentially confounding effect of lower performance on ERP results in the ASD group. Additionally, the high discrimination performance for autistic participants illustrates that the cognitively able participants in the autistic group under investigation here focused their attention on the task just like the NT group. In the following sections, we will discuss the role of typicality as well as category boundaries and the level of categorization as indexed by ERPs.

### Role of task characteristics during ultra-rapid semantic categorization

We determined the earliest evidence of semantic categorization in the EEG signal by using the difference wave between target and non-target images. Like Thorpe et al. [64], we observed a frontal modulation of the difference wave after 150 ms in NT adults, although restricted to the short presentation time and the food category. By contrast, the corresponding effect comparing animals and non-animals was located at frontal and occipital ROI in the original publication [64]. A number of modifications in our paradigm may have contributed to this result:

1. Stimuli: The original non-animal images were natural pictures (e.g., forests, mountains, lakes, buildings, flowers, and fruits; [64]), whereas in the present study, pictures of man-made objects were presented. Prior work has shown a different onset of neuronal responses to artificial vs. natural pictures [27], and thus, we cannot exclude that our results may reflect our choice of stimulus material.
2. Presentation times: The temporal characteristics of the difference wave are influenced by differences in the rate of presentation (i.e., 23.5 ms and 82.3 ms), as the effect was restricted to the short presentation rate which corresponds closely to the one used by Thorpe et al. [64]. We extended these original findings by demonstrating no effect of presentation time.
3. Response requirements: In contrast to the go/no-go task used by Thorpe and colleagues, in our paradigm, participants were asked to press a button after each image. Thus, we eliminated the need for motor inhibition of non-targets, which requires cognitive control processes along with the cognitive processes recruited for categorization.

For cognitively able autistic individuals, we also observed divergences for the animal category at frontal recording sites, albeit for the longer presentation duration. Likewise, peak amplitudes were larger for the animal category in the dN150 for cognitively able autistic individuals. Together, these findings suggest that the clinical population relied on different cognitive processes to complete the task.

### Similar effects for less distinct category boundaries for both groups

We found that less distinct category boundaries affect reaction times and performance both in cognitively able autistic and in NT adults, with longer reaction times and less accurate discrimination for food images, where boundaries between targets and non-targets were generally less distinct. The faster and more accurate categorization of animal images is in line with literature suggesting that the recognition of animals is based on various mid- and high-level visual features, for instance their global outline [34], diagnostic animal parts including the eyes, mouth, and limbs [12], and intermediate curvilinear features [73], together with the anatomical segregation of various category-selective neurons in the ventral temporal cortex [33, 68]. In line with behavioral results, larger difference between categories were observed for both groups in term of peak amplitude of the dN150, regardless of presentation time. Similarly, we observed smaller P2 amplitudes in both groups in response to food images compared to animal images, again reflecting higher task difficulty with the former set of stimuli. In fact, P2 amplitude has been shown to correlate inversely with task difficulty in general [8]. Our results thus suggest that ultra-semantic categorization is more difficult when objects belong to a category with fuzzier boundaries. Note that for the cognitively able autistic adults, this effect was only observed in the atypical condition, suggesting that the effect of category boundaries is only apparent in the more difficult condition. Additionally, we also found a more negative N2 for food stimuli than for animal stimuli in both groups (only trend in autistic group), replicating the findings of Antal et al. [3], who also found more negative deflections of the N2 component for non-animals compared to animal stimuli.

### Typicality effects in ASD

Previous studies showed that cognitively able autistic adults have difficulties with the outer edges of a category. This is important since most natural categories cannot be distinguished by simple features, but rather by typicality structures [21]. In other words, categorizing atypical images requires a more detailed level of categorization and thus needs longer and is less accurate [26]. In the present paradigm, both NT adults and cognitively able autistic adults showed comparable effects of typicality in behavior, with answers to atypical images being slower and less correct. In line with Vanmarcke et al. [66], we found no group differences in mean RT and d′. To substantiate our behavioral findings, we assessed the influence of typicality on the P2 and P3 components and observed a typicality effect in P2 for NT and in P3 for autistic adults. Whereas the P2 component reflects categorizing targets based on simple features, the P3 reflects categorizing targets based on complex, more arbitrary features. Note that both processes are interdependent, since categorization is based on either simple or more complex arbitrary features, as evidenced statistically by the presence of one effect with the concurrent absence of the alternate effect. This difference in target discrimination is also reflected in the timing of their occurrence. NT adults displayed a more positive P2 component for atypical images, whereas cognitively able autistic adults showed a more positive P3 component for typical images. These findings suggest that atypical images are more likely to be categorized based on their simple features by NT adults and based on arbitrary features by cognitively able autistic adults. There are two caveats in this interpretation of our findings. First, since typicality was assessed and rated by an independent sample of NT adults, the apparent difference in the response to or processing of typicality itself may actually reflect differences in the individual representation of typicality. So far, there is no study that addresses this issue. Instead of being rated by an independent group of NT adults, in future research, it would be valuable to measure typicality ratings by cognitively able autistic adults. This approach could shed light into the question of whether typical vs. atypical items are defined differently in ASD. As a second limitation, we should also consider that atypical images with perceptual similarity to typical images tend to be categorized as quickly as typical images, leading to an underestimation of typicality effects. Notwithstanding, we did observe slower responses to and less accurate discrimination of atypical images in both groups, and a qualitative different patterns of findings in the P2 versus P3 for each group, substantiating our approach. Since our results are the first to investigate these five factors — i.e., typicality effects in categories with different distinctiveness of their category boundaries in ultra-rapid categorization with different presentation times in cognitively able autistic and NT adults — concurrently using EEG, further information is needed to make elaborate statements about distinct cognitive processing in ASD. We want to encourage further investigations on this topic.

### Level of categorization

Visual input can be categorized on three hierarchical semantic levels — superordinate, basic, and subordinate level. Categorizing visual input on a lower level, i.e., in more detail, depends on the time available to perceive images [6]. In the present study, when an image was presented for a longer duration, participants responded faster and more accurately,a longer duration of visual presentation was associated with a slightly earlier N1 latency in the ASD group and a larger N2 amplitude in the NT group. The N1 component reflects differences between subordinate and basic level categorization [63], which we did not observe in NT adults. Together, these results suggest that cognitively able autistic adults use additional presentation time to categorize images on the subordinate level, even though they engaged in a superordinate level categorization task. However, interpretations of the N1 need to be taken cautiously, since we did not specifically control for multiple low-level visual properties (for instance, color parameter, visual complexity, luminance energy, and spatial frequency composition). Note that the observed difference in latency is numerically small but might still be of consequence since small changes early in the stream of cognitive processes might potentially have an impact on later cognitive processes.

In NT adults only, we found larger N2 amplitudes for the long presentation time, which may index object recognition [69], suggesting that NT participants used the additional presentation time for qualitatively different cognitive processes relative to a shorter presentation time. For autistic individuals, this comparison appeared as a trend, tentatively suggesting increased variability in cognitive processing when more time was available. In sum, in cognitively able autistic adults, longer presentation times lead to more detailed processing of images, supporting the notion of enhanced visual discrimination in cognitively able autistic adults as suggested for standard viewing times.

In addition to the classic triad of impairments, qualitative differences in visual processing have been consistently described over the last decades in autistic individuals [51]. The enhanced discrimination ability is shown, for example, by the fact that apparently minor changes in the environment, which are overlooked by NTs, are noticed by autistic individuals [49]. Enhanced discrimination abilities in ASD may contribute to a difficulty and/or reluctance in forming a prototype [51], supporting the notion that cognitively able autistic individuals prefer to categorize on a rule-based system rather than using prototypes [44].

### Disentangling the impact of interdependent factors on semantic categorization in ASD

In the present study, we demonstrated how each of three factors, category, typicality, and presentation time, together modulate ultra-rapid semantic categorization in autistic individuals. However, we need to acknowledge that additional factors are likely to have an impact on semantic categorization as well. For instance, as mentioned above, in the current study, we only investigated male participants. Hence, we cannot derive any conclusions or make any claims about autism spectrum in females. Since only few female participants in a gender-mixed group could not be validly assessed statistically, this has been one standard approach in this line of research, with obvious advantages in particular when moderate samples of autistic individuals are under investigation. We would like to take this opportunity to acknowledge that multiple issues remain open regarding female autistic populations. Fortunately, more recent investigations begin to address these important issues; there is an ongoing debate about sex and gender differences in autistic individuals, especially since autistic individuals have lower gender identification than NTs [11] and female autistic individuals camouflage more of their symptoms than their male counterpart [24, 59]. Studies indicate that adult female autistics have more lifetime sensory symptoms, fewer current socio-communication difficulties, and more self-reported autistic traits [32] as well as have restricted and repetitive behaviors and interests which are qualitatively and quantitatively unique in females [43]. Therefore, potential differences in categorization between male and female autistics remain to be established and disentangled from moderating and/or mediating factors like severity of autistic symptoms or linguistic abilities. Although challenging on the level of individual studies, it is crucial that potentially interdependent factors be investigated together in order to shed light on interaction effects. Since it is not feasible to assess all relevant factors in a single study, a careful balance is needed between disentangling several interdependent processes and holding other factors constant to maintain relatively homogenous groups of participants.

## Conclusion

Our results regarding cognitive processes during ultra-rapid semantic categorization may reflect findings of the social, communicative, and behavioral characteristics of ASD. Enhanced discrimination abilities could interfere with categorization, which depends on the ability to treat some object features within a category as similar despite perceptual dissimilarity [61]. Our results suggest that autistic individuals categorize images based on complex features, rather than based on simple features as NT adults do. The use of complex features to categorize images is an indication of enhanced discrimination abilities. Together with the difficulty of categorizing based on similarity [30], it may contribute to the preference for categorizing in a clear-cut, rule-based system in cognitively able autistic persons. Furthermore, longer N1 latencies suggest that cognitively able autistic individuals examined images in greater detail than NT adults when they had the opportunity. However, we can assume that the use of this additional information has no advantage on performance, based on our behavioral data demonstrating that cognitively able autistic adults did not outperform NT adults, at least under the task parameters employed here. Hence, a more detailed examination of objects might be used without any noticeable positive effect on their behavior. Of course, we concede that the heterogeneity of behavioral manifestations of ASD as well as the relatively modest sample size may mask or underestimate existing group differences in the current investigation. Moreover, we only assessed cognitively able autistic individuals who might engage in compensatory strategies.

Having more detailed or hyper-specific categorical representations means that the similarities between objects decrease, while the number of critical features for categorical judgments increases. Hyper-specific categorical representations, regarding social settings, would result in a reduced perception of similarities between situations and social cues. Similarities between complex perceptual inputs that vary on a number of dimensions are required to recognize to correctly understand social cues [42, 58]. Hence, communication skills and reciprocal social interaction might be more difficult for autistic individuals. Furthermore, the ability to quickly categorize is important to reduce the demands on memory capacity, releasing resources to focus on more pragmatically important aspects of our environment [21]. However, if not enough resources are available to focus on these other aspects of the environment, individuals on the autism spectrum will either feel overwhelmed or miss out on these aspects altogether, both of which are observed in ASD. Hence, it is necessary to investigate categorical representations and categorization of autistic individuals in more detail, to better understand their communicative skills and reciprocal social interactions as well as their restricted, repetitive, and stereotyped patterns of behavior [2].

## Data Availability

The datasets used and analyzed during the current study as well as a list including all statistical effects are available from the corresponding author on reasonable request.
